# The effect of a heel-unloading orthosis in short-term treatment of calcaneus fractures on physical function, quality of life and return to work – study protocol for a randomized controlled trial

**DOI:** 10.1186/s13063-019-3447-8

**Published:** 2019-06-04

**Authors:** Hagen Schmal, Anders Holsgaard Larsen, Lonnie Froberg, Julie Ladeby Erichsen, Carsten Fladmose Madsen, Lasse Pedersen

**Affiliations:** 1 Department of Orthopedics and Traumatology, Odense University Hospital, Sdr. Boulevard 29, 5000 Odense C, Odense, Denmark; 2 Department of Clinical Research, University of Southern Denmark, Odense, Denmark; 3 OPEN, Odense Patient data Explorative Network, Odense University Hospital, Odense, Denmark

**Keywords:** Calcaneus fracture, Aftercare, Study protocol, Ankle foot orthosis, Rehabilitation

## Abstract

**Background:**

There are no standardized therapy guidelines for rehabilitation of calcaneus fractures. While there is consensus on non or partial weight-bearing, the use of supporting devices such as specific foot ankle orthosis is still a matter of debate. Recently, a heel-unloading orthosis (“Settner shoe”) was introduced for aftercare of these fractures, allowing walking by shifting the load to the middle-foot and forefoot. This orthosis enables early mobilization of patients suffering from either one-sided or two-sided fractures. The Settner shoe can be applied in non-operative therapy and after surgery. Specifically in calcaneus fractures, early regain of physical activity has been highlighted as one of the key factors for quality of life and the ability to return to work. Thus, we hypothesize that mobilization with the Settner shoe results in improved quality of life and greater physical activity within the first 3 months.

**Methods:**

This is going to be analyzed by a randomized controlled study comparing treatment with and without this specific orthosis. The secondary outcome measure is the time point of return to work in patients aged between 18 and 60 years, with calcaneus fracture. Furthermore, the American Orthopaedic Foot and Ankle Society (AOFAS) ankle-hindfoot score, a 3-dimensional gait analysis, and the Euroqol-5 dimension-3 level (EQ-5D-3 L) questionnaire for quality of life are assessed.

**Discussion:**

This is the first trial applying a standardized rehabilitation protocol in patients with calcaneus fractures, aiming to improve the non-operative part of treatment by use of an orthosis.

**Trial registration:**

ClinicalTrials.gov, NCT03572816. Registered on 27 July 2018.

**Electronic supplementary material:**

The online version of this article (10.1186/s13063-019-3447-8) contains supplementary material, which is available to authorized users.

## Background

### Introduction and rationale

In the past, the scientific focus in the treatment of calcaneus fractures was the choice of operative or non-operative treatment. Although the evidence is ambiguous [1], recent meta-analyses suggest that surgery is associated with a higher likelihood of resuming pre-injury work and to reach a higher level of physical function and fewer problems when wearing shoes. However, non-operative therapy involves significantly fewer complications and infections [2, 3]. Typically, aftercare includes non or partial weight-bearing, but protocols differ and are often non-specific. In fact, there are no published studies comparing different procedures or special supporting devices. Recently, a heel-unloading orthosis (“Settner shoe” [4]) was introduced in the aftercare of calcaneus fractures, allowing walking by shifting the load to the middle-foot and forefoot. This orthosis does not only enable early mobilization of patients suffering from one-sided fractures, but also permits mobilization after two-sided fractures, avoiding the otherwise necessary use of a wheelchair. The Settner shoe can be applied in non-operative therapy and after surgery. Specifically in calcaneus fractures, early regain of physical activity has been highlighted as one of the key factors for quality of life and the ability to return to work [2, 3]. Thus, we hypothesize that mobilization with the Settner shoe results in greater physical activity within the first 3 months and improves the ability of patients aged 18–60 years to return to work after calcaneus fracture. Further outcome criteria are the American Orthopaedic Foot and Ankle Society (AOFAS) ankle-hindfoot assessment, a 3-dimensional gait analysis, and the Euroqol-5 dimension-3 level questionnaire (EQ-5D-3 L). This is the first trial applying standardized aftercare in patients with calcaneus fractures, aiming to improve non-operative treatment. Furthermore, the trial clarifies whether the economic cost of the equipment is scientifically justified.

### Objectives

We hypothesize that mobilization with the Settner shoe results in greater physical activity within the first 3 months after calcaneus fracture.

### Research questions

Does the application of a heel-unloading orthosis (Settner shoe) in patients with calcaneus fracture improve the following outcomes independent of operative or non-operative therapy:

1. Physical activity (minutes per day of activity)
2. Quality of life (EQ-5D-3 L)
3. Foot function (AOFAS)
4. Time necessary for return to work in patients between 18 and 60 years of age

### Trial design

The study design is a parallel group, randomized controlled trial with open allocation including all patients with calcaneus fractures independent of the type of initial therapy. It is an investigator-initiated trial.

## Methods/design

### Study setting

The protocol follows the SPIRIT guidelines as provided in Addional file 1. All patients treated for calcaneus fracture (DS920* *Fraktur af hælben* according to the Danish SKS-browser) at the University Hospital Odense are prospectively screened for eligibility and included in the trial if they fulfil the inclusion criteria as listed subsequently (see “Inclusion criteria”). The follow-up period is 6 months.

### Eligibility criteria

In Denmark, the treatment of calcaneus fractures is regarded as highly specialized, wherefore both decision-making and operations are performed for the whole Region of Southern Denmark in the Department of Orthopaedics and Traumatology at the University Hospital Odense. Therefore, patients are screened by consultants in the local trauma section.

#### Inclusion criteria

The inclusion criteria are:

- Fracture of the calcaneus, which is classifiable according to the Sanders’ classification (excludes avulsion fractures)
- Ability to understand Danish or English and answer the questionnaires
- Informed consent
- Age between 18 and 65 years

#### Exclusion criteria

The exclusion criteria are:

- Pathological fractures including fractures associated with Charcot foot
- Immature skeletal system
- Other fractures with influence on weight-bearing
- A soft-tissue condition that prevents the application of the Settner shoe within 3 weeks after treatment

### Interventions

After surgery or the decision to perform non-operative therapy, the schedule for the two groups are defined as follows:

1. Group 1 (treatment without the Settner shoe)
  Mobilization without weight bearing for 6 weeks starting with the day of the decision to apply non-operative therapy or open reduction and internal fixation; if needed a cast or other type of orthosis such as a static walker are applied; after the first 6 weeks weight-bearing is increased to 15–20 kg for another 4 weeks, then followed by 2 weeks with 35–45 kg; after that transition is made to full weight-bearing (always only if possible).
  Radiography after 6 and 12 weeks; depending on the results, the schedule for weight-bearing may be adjusted as necessary in the case of delayed healing or complications related to implants.
2. Group 2 (treatment with the Settner shoe)
  Mobilization with the custom-made heel-unloading orthosis (Settner shoe) without pads for 6 weeks, then 2 weeks with one pad, 2 weeks with 2 pads, and 2 weeks with 3 pads; after that full weight-bearing without any support (always only if possible).
  Radiography after 6 and 12 weeks; depending on the results, the schedule for weight-bearing may be adjusted as necessary in the case of delayed healing or complications related to implants.

Patients in both groups are informed about the aftercare at the time point of inclusion into the study.

### Outcomes

#### Overview of assessments

**Fig. 1:**
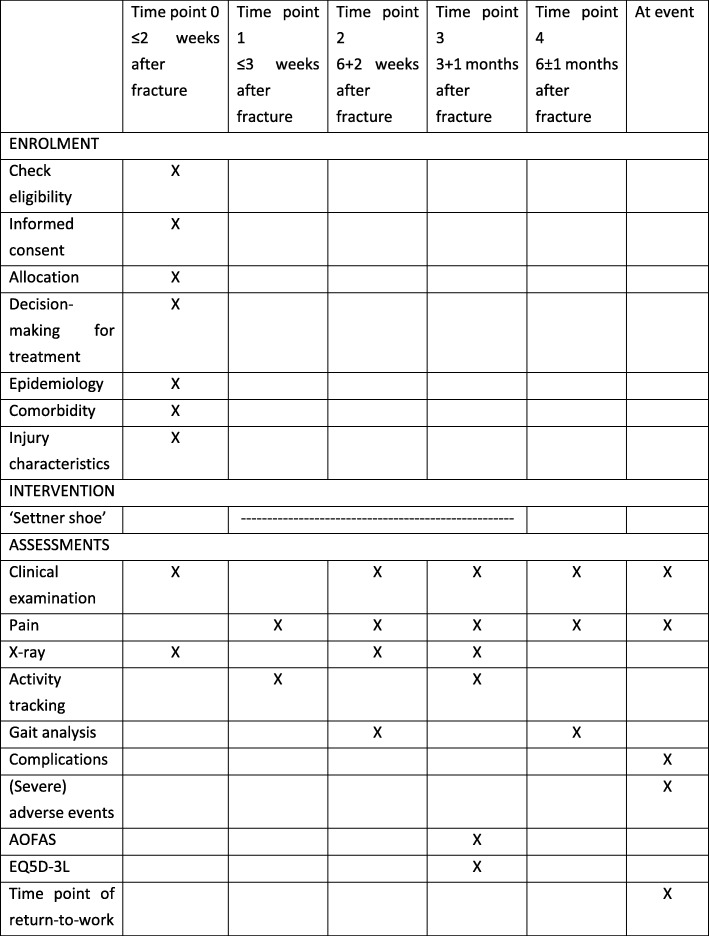
Schedule of enrolment, interventions, and for assessment of the different variables. AOFAS, American Orthopaedic Foot and Ankle Society; EQ 5D-3 L, Euroqol-5 dimension-3 level questionnaire

The schedule of enrolment, interventions, and assessments for the different variables is summarized in Fig. 1.

#### Primary outcome criterion

The EuroQol-5D-3 L questionnaire [5] at 3 months (time point 3) is the primary outcome criterion.

#### Secondary outcome criteria

The secondary outcome criteria are:

- Active minutes per day at time point 1 and 3
- Portion of highly active periods per day at time point 1 and 3

#### Exploratory outcome criteria

The exploratory outcome criteria are:

- AOFAS (time point 3) [6].
- 3-Dimensional gait analysis (time point 2 if possible, and timepoint 4).
- Time point of return to work of patients between 18 and 60 years of age.
- Range of motion (ROM): pronation and supination (percent of healthy side or assumed normal mobility) are analyzed as part of the clinical examination.
- Pain: the evaluation includes medication data and the visual analogue scale (VAS) score.
- Comparison between patients treated operatively and non-operatively

### Patient characteristics other than the outcome criteria

#### Epidemiology

The following characteristics will be recorded:

- Age
- Sex
- Body mass index
- American Society of Anesthesiologists (ASA) physical status classification system

#### Injury characteristics

The following characteristics will be recorded:

- Classification according to Sanders [7].
- Classification of open/closed tissue damage [8].
- Polytrauma (Injury Severity Score (ISS) ≥ 16, multiple injuries, mono-trauma).
- Indication for operation (dislocation, broadening, flattening of Böhler angle etc.) or conservative therapy (comorbidity, smoking etc.).
- Time between occurrence of fracture and surgery or start of non-operative therapy.

#### Healing and treatment characteristics

The following characteristics will be recorded:

- Bone healing after 3 months: union or delayed healing. The decision is made based on conventional radiographs including the clinical description of symptoms.
- Weight-bearing after 3 months: is it possible or not?
- Hindfoot axis (varus/valgus deformity, Böhler angle, subtalar osteoarthritis). The decision is made based on conventional radiographs and clinical evaluation.
- Type of operation (approach, fixation method, graft).

#### Complications

The following complications will be recorded:

- Infection (I).
- Deep venous thrombosis (DVT).
- Nerve paresis/palsy/disturbed sensibility (N).
- Subtalar posttraumatic osteoarthritis (PTOA) assessed using the Kellgren-Lawrence score [9].
- Subtalar instability (SI), clinically assessed.
- Local mechanical irritation by plate/screws (LI).
- Wound irritations (WI) as superficial healing problems or skin edge necrosis.
- Irritations associated with the use of the custom-made heel-unloading orthosis (Settner shoe).

### Participant timeline

An overview of the timeline is provided in Fig. 1. The clinical and radiological controls correlate with the usual guidelines for treating calcaneus fractures at the Odense University Hospital.

### Sample size and power analysis

The sample size calculation was based on data from a pilot study suggesting a 33% difference in quality of life (QoL) and activity, correlating with a clinically relevant difference. However, the 95% confidence interval was narrower for the EuroQol 5D-3 L questionnaire compared to all activity measurements. Therefore, QoL was chosen as the primary outcome measure. The desired ratio of group 2 to group for the sample size was set at 1 (i.e. the sample sizes are equal), the power at 80% and the two-sided confidence interval at 95%, calculated using the following equations [10]:

$$ {n}_1=\frac{\left({\sigma}_1^2+\frac{\sigma_2^2}{\kappa}\right){\left({z}_{1-\frac{\alpha }{2}}+{z}_{1-\beta}\right)}^2}{\Delta ^2} $$

$$ {n}_2=\frac{\left(\kappa \ast {\sigma}_1^2+{\sigma}_2^2\right){\left({z}_{1-\frac{\alpha }{2}}+{z}_{1-\beta}\right)}^2}{\Delta ^2} $$

where n_1_ = sample size of group 1, n_2_ = sample size of group 2, σ_1_ = standard deviation of group 1, σ_2_ = standard deviation of group 2, Δ = difference in group means, κ = ratio = n_2_/n_1_, Z_1-α/2_ = two-sided Z value (e.g. Z = 1.96 for 95% confidence interval), and Z_1-β_ = power.

The calculation resulted in 28 patients in each group. Allowing for a 10% drop-out rate, 31 patients will be included in each group.

### Recruitment

All consultants in the trauma section of the Department of Orthopaedics and Traumatology at the University Hospital Odense are involved in the recruitment process of the study.

## Methods: assignment of interventions (for controlled trials)

### Allocation

In the period between treatment (when the decision is made to opt for non-operative therapy or open reduction and internal fixation) and consolidation of the soft-tissue situation, the patients are randomized to aftercare with or without the custom-made heel-unloading orthosis (Settner shoe). As soon as the soft-tissue condition allows the adjustment, the patients are referred to an orthopaedic shoemaker, who equips the patient accordingly. Patients are randomized using the tool provided by the Odense Patient data Explorative Network (OPEN).

### Blinding (masking)

The allocation is not blinded, because it will be obvious to the patient, whether a special shoe is worn or not. Furthermore, the treating trauma surgeon needs to initiate the prescription.

## Methods: data collection, management, and analysis

### Management and collection

Research Electronic Data Capture (REDCap™), a secure application for online surveys and databases, is used to facilitate data management. The University Hospital Odense is an institutional partner of REDCap™, which was especially designed for biomedical research and fulfills all necessary safety features. This is supported by the OPEN initiative. REDCap™ used with the OPEN platform also supports a randomization function, which is used for the study. This agreement was approved by OPEN (project number 656, 18/29801).

### Statistical methods

Normally distributed numeric data sets are compared using the paired Student’s *t* test. Otherwise or in the case of non-numeric data, nonparametric tests such as the Mann-Whitney U test for two variables and the Kruskal-Wallis H test for multiple variables are used to determine the significance of any difference. Spearman’s ρ is calculated to test correlation. Incidence is compared using the chi square test. Binary outcomes are analyzed by logistic regression including at the least the a priori confounders age and sex. Considering the fact that the ability of patients to mobilize after injury is highly dependent upon pain and swelling, which in turn is partly dependent on fracture type and treatment modality, special attention is paid to the classification according to Sanders, which will be tested as one of the most influential confounders in addition to the type of treatment. Continuous variables will be analyzed by multiple regression; both types of regression analysis include confounders identified by dichotomous calculations. Other variables to be included are epidemiological parameters classification, and treatment modality. Data will be analyzed using the intention-to-treat approach, which means that patients randomized to the orthosis group are considered to have used the orthosis.

## Methods: monitoring

### Data monitoring

A data manager was assigned to the study by OPEN, who supports the data coherence. Furthermore, the study will be monitored yearly by a study nurse, who is assigned to the project.

### Harms

Any adverse or severe adverse events will be registered during the trial. In particular, expected adverse events are local mechanical irritations, which are listed in the section for registration of patient characteristics in addition to the outcome criteria.

## Ethics and dissemination

### Ethics

The study was approved by the ethical board of the Region of Southern Denmark (https://komite.regionsyddanmark.dk, S-20170219).

### Patient law/privacy

All data collected from patients are protected by the Act on Processing of Personal Data and Health Act according to Danish Data Protection Agency. The project was reported to the local data safety authorities (*Datatilsynet* under *Regionens Paraply* 2012-58-0018, 17/44501). The database was established with OPEN; however, other possibly related files are stored in a secure Sharepoint (https://secure.regionsyddanmark.dk/projektrum/ProTibExPla/SitePages/Startside.aspx).

### Publication

Reporting will be conducted according to Consolidated Standards of Reporting Trials (CONSORT guidelines (http://www.consort-statement.org/) and published in a peer-reviewed journal. The authorships are granted according to the International Committee of Medical Journal Editors (ICMJE) guidelines.

## Trial registration

The project was registered and approved by the Ethical Board Region Southern Denmark (Project-ID S-20170219). Furthermore, the project was approved according to the Act on Processing of Personal Data (Journal number 17/44501). Moreover, the trial was registered at ClinicalTrials.gov (Identifier NCT03572816).

## Access to data

LP and HS will have access to the final trial dataset and will perform the final analysis. This is monitored by the assigned data manager (OPEN). Access to the data is regulated by the contract with OPEN.

## Appendices

### Measurement of physical activity

For monitoring of activity, the patients are equipped with an Axivity™ AX3 (Newcastle upon Tyne, UK) at the lateral femur of the unaffected side. If there is a fracture at both sides, the trackers are attached to the side with the less complicated fracture. The wear time is 7 days. At day 7 ± 14 (discharge, time point 1) and after 3 + 1 months (time point 3) patients are equipped, and activity signals are analyzed by calculating the mean of all acquired 24-h data during these two periods. Regarding the Axivity™ AX3, the portion of highly active periods per day and the number of active minutes, correlating with walking activity, have shown to be the most discriminating in the validation study [11]. Therefore, these parameters are selected. Reliability is checked by wear-time analysis based of change in signal vector magnitudes and temperature monitoring. Correlating with these two data points, the EuroQol 5D-3 L questionnaire [5] and the AOFAS [6] are monitored.

### Orthosis

The heel-unloading orthosis (Settner shoe [4]) can be used for conservative treatment and aftercare following surgery. It needs to be assembled from pre-formed and size-adjusted parts. This is done by an orthopedic shoemaker. The increased weight-bearing is achieved by pads. The shoe is worn without pads for the first 6 weeks, then the first pad is applied. Every 2 weeks a further pad is put in the shoe, which is removed after 3 months. Preliminary data suggest that this heel-unloading orthosis reduces the number of days of inability to work.

### Three-dimensional gait analysis

All outcome calculations will be based on measurements taken during gait using a 3D Vicon MX movement analysis system with eight cameras operating at 100 Hz (Vicon, Oxford, UK) and two AMTI force-plates (AMTI, OR6–7, Watertown, MA, USA) embedded at floor level, operating at 1000 Hz. A technician experienced in gait analysis and the Vicon system will attach reflective markers that reflect infrared light according to the Vicon Plug-in-Gait marker set and model [12].

### Patient involvement

The patients give feedback about the actual use of the orthosis including the possible benefit associated with its application. Furthermore, activity measurements provide objective data about patient mobility during early aftercare and signal feedback to the patient. By this, the patients themselves can postoperatively control their increasing activity. This includes a scientifically supported evaluation by the study investigators at the defined follow-up time points at 6 weeks and 3 months after start of therapy.

### Consent form

The consent form used during the trial is provided as Additional file 2 and 3 in Danish and English.

## Discussion

This manuscript presents a protocol for a standardized rehabilitation protocol in patients with calcaneus fractures, aiming to improve the non-operative part of their treatment by use of an orthosis. Aftercare in fracture treatment appears to be as important as the operation itself and is in case of non-operative treatment the actual therapy. Recently, it was shown that early functional exercise and weight-bearing activity can smooth and shape the subtalar joint following intraarticular calcaneus fractures “and reduce the residual displacement of the articular surface, improving functional recovery of the affected foot” [13]. Therefore, early rehabilitation should be optimized and implemented in clinical practice. There are many different factors and parameters, which can be influential and have not yet been evaluated in a standardized manner. An article describing the different rehabilitation possibilities for a single calcaneus injury compared to calcaneus fractures in patients with multiple injuries surprisingly concluded that there were no differences in outcome measures between these two groups [14]. While orthoses are in the focus of non-operative therapy in foot-related diseases [15], the use of aids and supporting devices in the treatment of fractures is often totally neglected. Usually, short rehabilitation protocols are provided without too much into detail [16]. Therefore, reliable protocols evaluating the use of the often applied orthotic devices appear to be just as necessary as a standardized procedure for the operation.

Pain, disability, and instability in the joint are the most important symptoms in patients with osteoarthritis or fractures, causing distress to many people [17]. Considering this, a quality of life score (EuroQol 5D-3 L questionnaire) was chosen as the primary outcome measure. Physical activity correlated with quality of life after proximal femur fractures, proximal tibia fractures and ankle fractures, however, the spread of data was higher than the confidence interval obtained with the EuroQol 5D-3 L questionnaire.

A major concern of the study is heterogeneity, including intra-articular fractures of all types and severity, and both operative and non-operative treatments. However, independent of the injury and the treatment, one group will start with weight-bearing (with the calcaneus free-hanging) and the other one without. The expectation is that weight-bearing is the factor that will make the difference in clinical outcome and not the intervention. Furthermore, the allocation is randomized, which hopefully secures even distribution of the influential factors. Moreover, special attention is paid to the classification according to Sanders, which will be tested as one of the most influential confounders in addition to the type of treatment.

Summarizing, the trial will contribute to elucidation of the value of orthosis treatment in the aftercare of calcaneus fractures. Considering the fact that some hospitals regularly use this equipment, the study can help to evaluate whether the economic cost is justified.

## Trial status

Protocol version: 2.6 from 17 December 2018.

The recruitment has not yet begun and starts in March 2019. Recruitment will presumably be completed in January 2021.

## Additional files

Additional file 1: SPIRIT 2013 Checklist: Recommended items to address in a clinical trial protocol and related documents*. (DOC 125 kb)

Additional file 2: Informed consent form in Danish. (PDF 17 kb)

Additional file 3: Informed consent form in English. (PDF 17 kb)

## Data Availability

Original data will be provided in an anonymized form together with the publication.

## Abbreviations

AOFAS: American Orthopaedic Foot and Ankle Society
ASA: American Society of Anesthesiologists
CALCFRO: Calcaneus Fracture Orthosis
DVT: Deep vein thrombosis
EQ-5D-3 L: Euroqol-5 dimension-3 level questionnaire
ISS: Injury Severity Score
OPEN: Odense Patient data Explorative Network
PTOA: Subtalar posttraumatic osteoarthritis
REDCap™: Research Electronic Data Capture
ROM: Range of motion
