# Supplementary material for: The effect of a heel-unloading orthosis in short-term treatment of calcaneus fractures on physical function, quality of life and return to work – study protocol for a randomized controlled trial
Source: Trials. 2019 Jun 4;20:324. doi: 10.1186/s13063-019-3447-8 (PMC6549309; doi:10.1186/s13063-019-3447-8)
Supplement: Supplementary file 2 — Informed consent form in Danish. (PDF 17 kb) [file 13063_2019_3447_MOESM2_ESM.pdf]

(S2)

Informeret samtykke til deltagelse i et sundhedsvidenskabeligt forskningsprojekt.

Forskningsprojektets titel: The effect of a heel-unloading orthosis in short-term treatment of calcaneus fractures on physical function, quality of life and return to work – a randomized controlled trial

Erklæring fra forsøgspersonen:

Jeg har fået skriftlig og mundtlig information og jeg ved nok om formål, metode, fordele og ulemper til at sige ja til at deltage.

Jeg ved, at det er frivilligt at deltage, og at jeg altid kan trække mit samtykke tilbage uden at miste mine nuværende eller fremtidige rettigheder til behandling.

Jeg giver samtykke til, at deltage i forskningsprojektet og har fået en kopi af dette samtykkeark samt en kopi af den skriftlige information om projektet til eget brug.

Forsøgspersonens navn: ____

Dato: ________________ Underskrift: ____

Hvis der kommer nye væsentlige helbredsoplysninger frem om dig i forskningsprojektet vil du blive informeret. Vil du **frabede** dig information om nye væsentlige helbredsoplysninger, som kommer frem i forskningsprojektet, bedes du markere her: __________ (sæt x)

Ønsker du at blive informeret om forskningsprojektets resultat samt eventuelle konsekvenser for dig?:

Ja _____ (sæt x) Nej _____ (sæt x)

Erklæring fra den, der afgiver information:

Jeg erklærer, at forsøgspersonen har modtaget mundtlig og skriftlig information om forsøget.

Efter min overbevisning er der givet tilstrækkelig information til, at der kan træffes beslutning om deltagelse i forsøget.

Navnet på den, der afgiver information:

Dato: ________________ Underskrift: ____

Projektidentifikation: (Fx komiteens Projekt-ID, EudraCT nr., versions nr./dato eller lign.)

61555
