# Supplementary material for: The effect of a heel-unloading orthosis in short-term treatment of calcaneus fractures on physical function, quality of life and return to work – study protocol for a randomized controlled trial
Source: Trials. 2019 Jun 4;20:324. doi: 10.1186/s13063-019-3447-8 (PMC6549309; doi:10.1186/s13063-019-3447-8)
Supplement: Supplementary file 3 — Informed consent form in English. (PDF 17 kb) [file 13063_2019_3447_MOESM3_ESM.pdf]

Ethical board / consent

(S2)

Informed consent for participation in a health care research project

Project's title: The effect of a heel-unloading orthosis in short-term treatment of calcaneus fractures on physical function, quality of life and return to work – a randomized controlled trial

Declaration by the participant / patient:

I have received written and oral information and I know enough about the purpose, method, advantages and disadvantages to say yes to participate.

I know it's voluntary to participate and that I can always withdraw my consent without losing my current or future rights to treatment.

I agree to participate in the research project and have received a copy of this consent sheet as well as a copy of the written information about the project for its own use.

Participant's name: ____

Date: _________________Signature: ____

If new important health information comes forward about you in the research project, you will be informed. Would you like to disclose information about new essential health information that appears in the research project, please select here:______________(set x)

Do you want to be informed about the results of the research project and any consequences for you?:

Yes_____(set x) No______(set x)

Declaration of the person providing information:

I declare that the subject has received oral and written information about the trial.

In my conviction, sufficient information has been provided for a decision to participate in the trial.

The name of the person providing information:

Date: _________________Signature: ____

Project identification: (i.e. EB project-ID, EudraCT nr., version nr./date or similar) 61555
